# Development of a Mannequin for Simulation-Based Trials Involving Respiratory Viral Spread During Respiratory Arrest and Cardiopulmonary Arrest Scenarios

**DOI:** 10.7759/cureus.20304

**Published:** 2021-12-09

**Authors:** Cindy Luu, Margaux Chan, Leo Langga, Elizabeth Bragg, Alyssa Rake, Caulette Young, Jennifer Lau, Edward Guerrero, Joshua Buan, Todd Chang

**Affiliations:** 1 Department of Emergency Medicine, Children's Hospital Los Angeles, Los Angeles, USA; 2 Las Madrinas Simulation Center, Children's Hospital Los Angeles, Los Angeles, USA; 3 Department of Respiratory Medicine, Children's Hospital Los Angeles, Los Angeles, USA; 4 Department of Anesthesiology Critical Care Medicine, Children's Hospital Los Angeles, Los Angeles, USA

**Keywords:** mannequin-based training, resuscitation quality, drape, patient barrier devices, covid-19

## Abstract

During the coronavirus disease 2019 (COVID-19) pandemic, mannequin models have been developed to mimic viral spread using fluorescent particles. These models use contraptions such as a spray gun or an exploding latex balloon to emanate a sudden acceleration of particles, simulating a “cough” reflex. No models have been developed to mimic passive aerosolization of viral particles during a cardiopulmonary arrest simulation. Our novel approach to aerosolization of simulated viral spread allows for a continuous flow of particles, which allows us to maintain components of high-fidelity team-based simulations. Our simulated model emanated GloGerm (Moab, UT) from the respiratory tract using a continuous nebulization chamber. Uniquely, the construction of our apparatus allowed for the ability to perform full, simulated cardiopulmonary resuscitation scenarios (such as chest compressions, bag-mask ventilation, and endotracheal intubation) on a high-fidelity mannequin while visualizing potential contamination spread at the conclusion of the simulation.

Positive feedback from users included the ability to visualize particulate contamination after cardiopulmonary resuscitations in the context of personal protective equipment usage and roles in resuscitation (i.e. physician, respiratory therapist, nurse). Negative criticism towards the simulation included the lack of certain high-fidelity feedback markers of the mannequin (auscultating breath sounds and checking pulses) due to the construction of the particle aerosolization mechanism.

## Introduction

The American Heart Association (AHA) has recently provided new guidance for resuscitation management in the face of the coronavirus disease 2019 (COVID-19) pandemic, with the goal of reducing exposure to virus aerosolization during bag-valve-mask (BVM) ventilation, endotracheal intubation, and cardiopulmonary resuscitation (CPR) [1-3]. While these recommendations address concerns regarding a healthcare team’s excess exposure to aerosolized particles, they could potentially have a negative impact on resuscitation quality.

Simulations are well-positioned to examine how potential viral spread occurs during resuscitation care that would otherwise have a high aerosolization risk for healthcare providers. However, most simulations prior to the COVID-19 pandemic had focused on cough simulators and environmental spread measurements in a laboratory setting [4-6]. More recent papers have described the use of continuous nebulization in addition to bag-valve-mask to replicate viral spread but do not offer the ability to perform simultaneous high-fidelity simulation [7].

During the pandemic, novel solutions to visualize viral spread have used either airborne particle measurements or fluorescent particles such as GloGerm (Moab, UT) under blacklight [8-11]. However, the airborne particle measurements are not optimal in a room with substantial human movement such as during a resuscitation. GloGerm is a non-toxic fine particle made of a plastic resin that is ultraviolet (UV) luminescent. Prior GloGerm simulator models have focused on a cough model, in which a burst of the fluorescent material is expelled once, or in a model that requires constant darkness and blacklight. This model differs from real-life cardiopulmonary arrest scenarios, in which the rooms are typically fully lit with potentially unseen aerosolized viral particles.

Therefore, our objective was to develop a mannequin-based simulator that enables simulated viral spread during resuscitation for respiratory arrest and cardiopulmonary arrest. Our ultimate goal was to use this type of mannequin for further simulation-based research to measure estimated simulated viral spread that could test interventions during a cardiopulmonary arrest scenario [12].

We had four requirements for the simulator: (1) a constant ‘emanation’ of GloGerm as opposed to a single cough or burst, activated and deactivated far away from the mannequin, (2) origination of simulated virus from the respiratory tract and naso-oropharynx, such that a mask with a tight seal would contain the spread, (3) the ability to be intubated with normal anatomy (e.g., no visible tubing/nozzles), and (4) the ability to perform chest compressions.

This technical report focuses on the construction and use of our mannequin during a cardiopulmonary resuscitation simulation, as well as barriers and limitations associated with its unique mechanism.

This article was previously presented as a meeting abstract at the 2021 American Academy of Pediatrics Conference, Section on Emergency Medicine on October 9, 2021.

## Technical report

Project design and implementation

Deconstruction and Construction of the Mannequin

**Figure 1:**
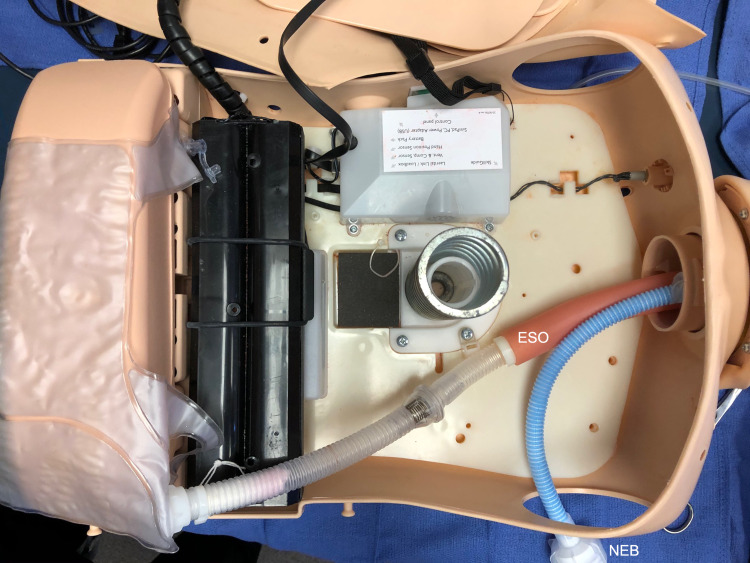
Deconstructing the mannequin The oropharynx component is detached from the stomach and trachea, and the Resusci Anne lungs are removed from the mannequin. ESO: esophagus, NEB: AirLife Misty-Max nebulizer with GloGerm powder

**Figure 2:**
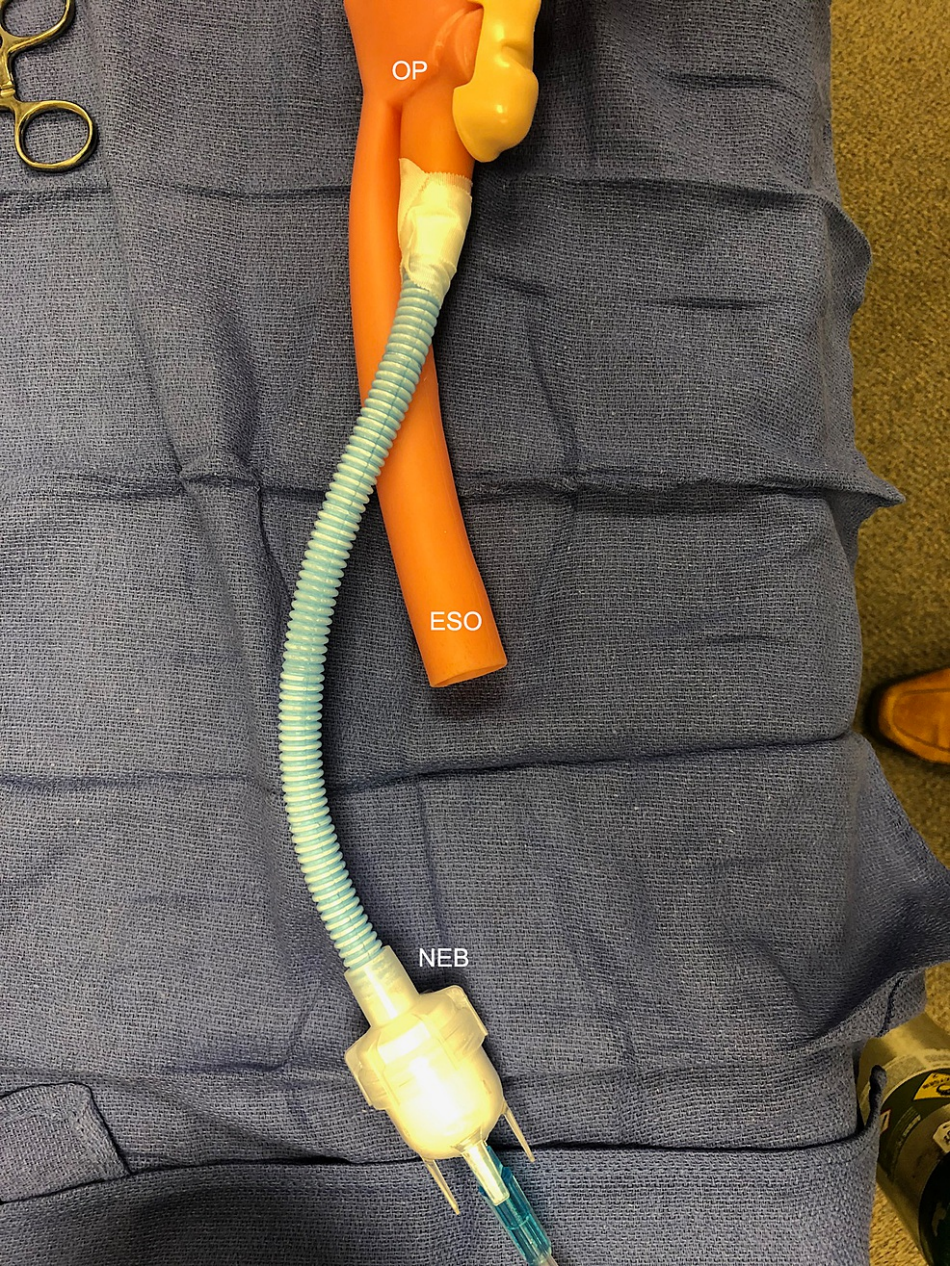
Attachment of nebulizer to the mannequin The AirLife Misty-Max nebulizer is connected via infant ventilator tubing that sits inside of larger ventilator tubing and is secured internally to the mannequin’s trachea. ESO: esophagus, NEB: AirLife Misty-Max nebulizer with GloGerm powder, OP: oropharynx

**Figure 3:**
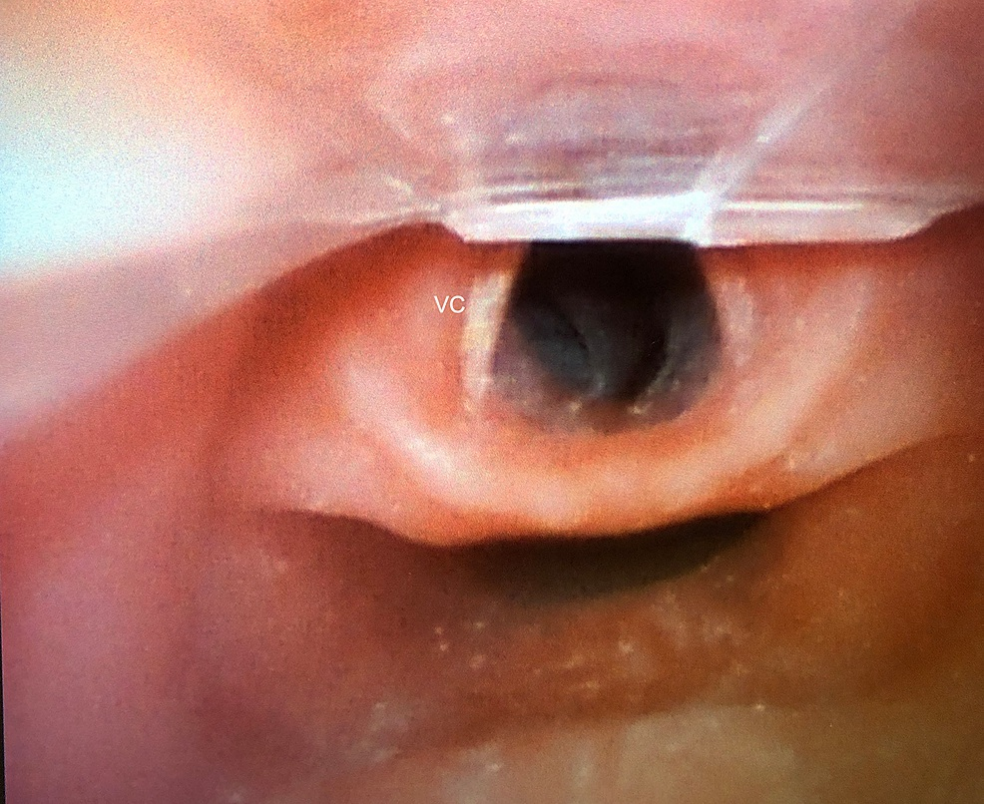
View of the oropharynx during intubation The terminal end of the AirLife Misty-Max nebulizer occurs distal to the vocal cords, allowing an unobscured view of the airway during intubation. VC: vocal cords

**Figure 4:**
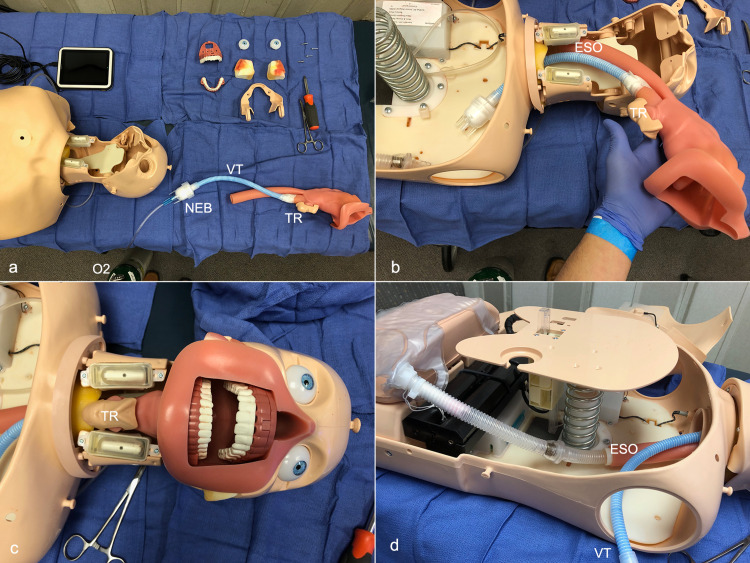
Construction of the mannequin 4a: Individual parts of the mannequin face and airway dissembled. Note that the AirLife Misty-Max nebulizer is attached to the trachea via infant ventilator tubing. 4b: Insertion of the trachea/esophagus into the mannequin facial structure. 4c: Facial structures placed back onto the mannequin. 4d: Chest plate for compressions secured back onto spring. The stomach reconnected to the esophagus. The nebulizer filled with GloGerm particles and will be placed upright inside the chest cavity. ESO: esophagus, NEB: AirLife Misty-Max nebulizer with GloGerm powder, O2: oxygen source, OP: oropharynx, TR: trachea, VT: ventilator tubing

A Resusci Anne intubatable QCPR mannequin (Laerdal, Stavenger, Norway) was modified for this simulated virus mannequin. First, the oropharynx component is detached from the stomach and trachea, and the ResusciAnne lungs are removed from the mannequin (Figure 1). The AirLife Misty-Max nebulizer is connected via infant ventilator tubing that sits inside of a larger ventilator tubing (Figure 2) and is secured internally to the mannequin’s trachea, with its terminating end sitting in the oropharynx without obscuring the laryngoscope view (Figure 3). The laryngoscopy view of the oropharynx is otherwise unchanged from the standard mannequin. The oropharynx and jaw are replaced, and the stomach is reattached. Because the Resusci Anne mannequin has armholes, the oxygen tubing from the nebulization chamber can be looped out via one of the armholes and attached to an oxygen (or air) source. The nebulization chamber is filled with GloGerm powder to capacity and positioned within the chest cavity of the mannequin. Figure 4 demonstrates how we deconstructed and constructed the mannequin.

Detecting GloGerm Powder

Cheng et al. describe two main types of simulation-based research: (1) studies that assess how effective simulation is as a training modality and (2) studies where simulation is used in itself as investigation [12]. The latter methodology allows us to investigate a safe clinical model to study how this simulator mannequin may affect the environment of the (i.e., particulate contamination during cardiopulmonary resuscitation simulations). Simulations were performed in-situ within various departments in the hospital (emergency room, medical-surgical units, and intensive care units). Participants were asked to complete a cardiopulmonary arrest simulation using our GloGerm mannequin, involving chest compressions, bag-valve-mask ventilation, and intubation. Participant teams included a minimum team of four members, including an intubating physician, respiratory therapist, bedside nurse, and medication nurse. Due to hospital-related COVID protocols, a maximum team of six members was allowed.

Each study group participated in one simulation scenario involving cardiopulmonary arrest in a known COVID patient, requiring chest compressions and intubation. Bag-valve-mask ventilation and intubation were performed during the scenario, with the use of a viral filter. At the completion of the simulation study, all participants were asked to freeze in place with all their personal protective equipment (PPE) on. The room lights were then turned off, and researchers examined each participant with a UV floodlight. Due to the small particle size, each participant was thoroughly examined with a UV light to record the presence of GloGerm particles on various aspects of their PPE and body. Resuscitation equipment used during the scenario (crash cart, laryngoscope, etc.), as well as contaminated areas in the room (door handle, floor, etc.) were also examined under UV light.

Evaluation and Feedback

At the conclusion of the simulation, we held a debrief session and had participants fill out course feedback. This course feedback was a five-point Likert scale to assess the overall simulation scenario, maintaining our standard educational debrief after simulations. We structured the debrief about both contamination and the resuscitation mechanics, requiring debriefers to prepare two fundamentally different but related topics. We then collected standard course feedback from participants via a form to rate the equipment and simulation.

Barriers and lessons

One of the first barriers we encountered in the design of this project was determining the correct methodology of aerosolizing an invisible, non-toxic, blacklight-fluorescent substance to mimic COVID-19 particles during simulation. We first attempted three substances using nebulization as a dispersion strategy (tonic water, GloGerm powder, GloGerm liquid) using nebulized aerosolization. Studies looking at the effect of suction-assisted laryngoscopy have shown that tonic water alone can be detectable via blacklight [13]. During our trial of using tonic water, we found it is difficult to see the tonic water depositing on surfaces and particularly on surfaces further away from the source.

GloGerm comes in multiple formulations - oil, liquid (mist), and powder. Majid et al. mixed GloGerm liquid with tonic water under 8 L/min to visualize aerosolization during percutaneous tracheostomy as a solution [14]. Patel et al. used a spray gun to disperse liquid GloGerm, which simulated a burst pattern similar to coughing [15]. Rather than ‘spraying’ the germ like a cough, our goal was to create a mannequin that emanated particles from the oropharynx passively. We had two aerosolization methods available in our clinical areas of practice: the AirLife Misty-Max nebulizer, commonly used for albuterol nebulization, and an Aerogen vibrating mesh nebulizer (Galway, Ireland). In either method, the GloGerm liquid formulation was too heavy to be appropriately aerosolized in a fine mist, and the fluorescent particulate matter remained trapped within the nebulization chamber and tubing. Similarly, tonic water could nebulize appropriately but did not sufficiently deposit onto surfaces for easy visualization. Even when the GloGerm liquid formulation was mixed with tonic water, the Glogerm particles were too heavy to aerosolize into a mist.

**Figure 5:**
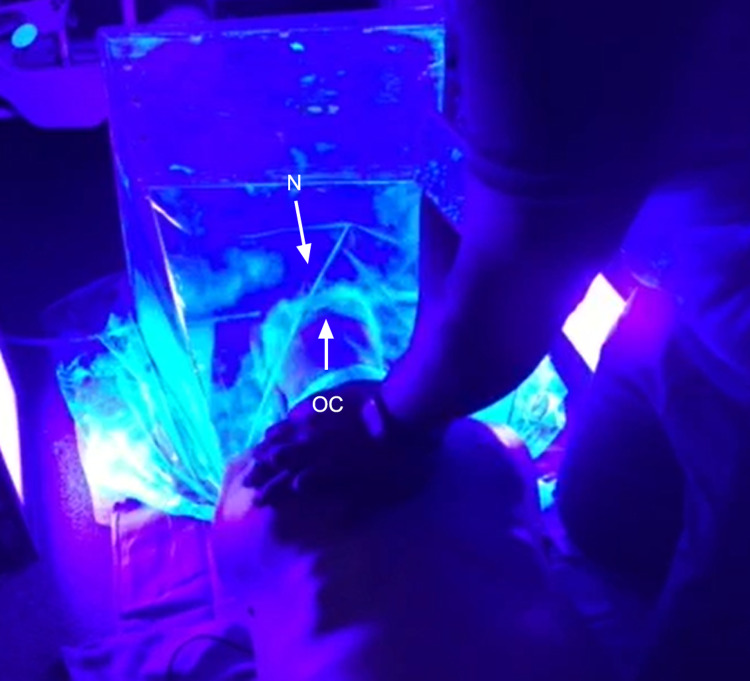
Performing chest compressions with our nebulized mannequin model under blacklight Note emanation of GloGerm from the nares and oral cavity. N: nares, OC: oral cavity

We then transitioned to GloGerm powder. Each powder molecule is 5 µm, fine enough to be expelled through a nebulization chamber. Several techniques have been described that allow the expulsion of powder through the mannequin using a manual bag-valve-mask or a syringe push [15]. Our goal was to have the particulate matter passively emanate from the mannequin during the entirety of the simulation. The GloGerm powder was filled within a Misty-May nebulizer chamber and seated upright within the torso of the mannequin. The nebulizer was then connected via infant ventilator tubing, sitting inside larger ventilator tubing, and secured internally to the mannequin’s trachea. The oxygen connecting tubing was then looped out via one of the mannequin armholes and attached to the flowmeter. The next barrier we encountered was determining an adequate flow rate to propagate the Glogerm powder from the oropharynx. After experimenting with various liters of oxygen flow, we determined that 6 L/min of flow per minute provided sufficient visualized aerosolization. When determining the optimal flow rate for the emanating simulated virus, we found that GloGerm powder in this mannequin configuration was intensely visible as a white powder at any flow greater than 7 L/min, even with no blacklight. Slower flow rates than 6 L/min had very minimal deposits even with resuscitations longer than 15 minutes. The rate of 6 L/min allowed the powder to emanate from the mannequin’s mouth but still remain ‘invisible’ without a blacklight to the participants. (Figure 5, Video 1).

**Video 1:** Demonstration of CPR and GloGerm nebulization under blacklight Video demonstrating the emanation of GloGerm powder from the oro-nasopharynx of the simulation mannequin under a black light, using 6 L/min of flow through a nebulizer, while performing chest compressions.

GloGerm powder, unlike liquid versions, does not stick well to certain plastic or acrylic surfaces such as face shields or a powered air-purifying respirator (PAPR) hood. Furthermore, occasionally small amounts of environmental dust can look like small deposits of GloGerm powder when under a blacklight. Unlike studies that use liquid GloGerm, the contaminated surfaces are relatively subtle and require careful visual scrutiny to see, particularly on clothing, protective equipment, and medical equipment [16].

Observations and feedback

We have observed 13 simulations using this mannequin set-up in the context of a randomized-controlled simulation-based descriptive study to evaluate the effect of patient barrier devices on resuscitation quality. Overall, participants are particularly impressed by the novelty of the simulator and the ability to use blacklight during debriefing to determine contamination. After visualizing the degree of contamination, debriefing sessions often focused on strategies in their routine clinical practice to mitigate cross-contamination.

We noticed substantial feedback about the lack of certain real-time features on the mannequin from a group of providers expecting features common to mid and high-fidelity simulations. This configuration removes the visual chest rise during positive ventilation because the GloGerm apparatus removes the lungs that inflate the chest wall of the mannequin. This required our simulations to have a substantial amount of pre-briefing, to indicate all of the elements of the mannequin that would not be available such as pulses and respirations. Alternative strategies to convey physical exam findings were required when participants performed respiration and pulse checks during the simulation because providers could not receive these high-fidelity cues directly from the mannequin. The simulator requires an independent vital signs monitor coupled with the scenario to provide vital sign changes, including apnea and pulselessness. This was accomplished with a separate, hidden high-fidelity mannequin with its vital signs, or with a vital sign simulator App (SimMon®, Castle+Anderson, Hillerød, Denmark).

In physiologic breathing, during inspiration, respiratory muscles contract to generate negative pressure to draw air into the lungs. During exhalation, respiratory muscles relax and positive pressure returns to the chest cavity to force air out of the lungs. The amount of air that moves in and out during this cycle, also known as the tidal volume, varies with effort and age. In a healthy adolescent male, an average tidal volume is roughly 500 milliliters, with a minute ventilation rate of 12 to 14 breaths per minute [17]. Minute ventilations subsequently range from 6 liters to 7 liters a minute for normal respiratory effort. In respiratory distress or apnea, this volume can dramatically increase or decrease accordingly.

While we ideally wanted this design to mimic physiologic breathing, this was limited by the construction of our mannequin. In removing the internal lung structure and connecting it to a continuous nebulizer, we allow for continuous airflow from the oropharynx, rather than changes to inspiratory and expiratory pressures associated with normal respiration cycles. Though we were not able to replicate these cycles, our GloGerm flow rate of 6 L per minute corresponds to the 6-liter minute ventilation of a healthy adolescent male. Additionally, we noted that without performing chest compressions on our mannequin, the GloGerm powder would reach a certain point where the powder would settle within the tubing and slow in its laminar flow. The intrathoracic pressure generated with each chest compression allows for the GloGerm flow to reinitiate in a pulsatile fashion corresponding with compressions. This, in addition, may contribute to greater emanation and particulate spread during chest compressions with CPR.

Data collection

Our simulations used a standard course feedback form to rate the equipment and simulation. Out of n = 43 respondents from 13 scenarios, the feedback for this mannequin embedded simulation is shown in Table 1. We collected overall feedback about the simulator and the use of simulated viruses and blacklight examination as part of a resuscitation scenario. In the context of a team resuscitation, no participant had prior experience with this type of simulator nor the use of blacklight following a pediatric advanced life support (PALS) algorithm scenario.

**Table 1:** Course feedback and evaluation using a 5-point Likert scale (n=43)

Likert Scale Survey Question: Strongly Disagree=1; Disagree=2; Neutral=3; Agree=4; Strongly Agree=5	Score
Pre-Brief	
There was a clear understanding of participants' expectations during the in-situ simulation	4.39
I was adequately introduced to the simulator and its capabilities	4.50
The simulation objectives were clearly defined	4.56
Scenario	
The subject matter content was presented clearly in the scenario	4.58
The simulation was realistic and made sense	4.34
The simulation scenario was applicable to my practice	4.67
The simulation was an appropriate amount of time	4.50
The facilitators allowed me to feel comfortable during the simulation	4.63

Negative feedback during debrief sessions held after completion of the simulation included how the mannequin set-up was relegated to a low fidelity nature with no palpable pulses, chest rise, or lung sounds.

Participants had overall positive feedback on the incorporation of the simulated virus release mechanism embedded in a typical in situ simulation, particularly during the early months of the COVID-19 pandemic in 2020. Participants felt that the objectives of the resuscitation were clearly defined (i.e., performing cardiopulmonary resuscitation as a team using personal protective equipment on a COVID-19 patient), and the simulator was useful in demonstrating the distant amount of viral particle spread in resuscitations.

## Discussion

We present a novel simulation method for visualizing viral spread using fluorescent particles during team-based cardiopulmonary resuscitation. We structured the debrief about both contamination and resuscitation methods, facilitating discussion on both human factors and contamination considerations. Some discussions focused on traditional human factors related to resuscitation (i.e., communication, availability of resources, appropriate cardiopulmonary resuscitation techniques) while other discussions focused more on the degree of contamination that was visualized after the simulation. While some participants felt protected from the contamination due to their PPE, other participants focused on strategies to further minimize future contamination.

Additional discussion focused on the loss of some high-fidelity components of the mannequin due to its construction (i.e., auscultating breath sounds or checking for pulses), which was dependent on the research team to display written signage during these steps of the resuscitation. Despite the loss of some high-fidelity components, our model was successful in the visualization of particulate spread after a cardiopulmonary arrest simulation. Unlike other models, our simulation allows for a slow continuous emanation of simulated virus from the oro- and nasopharynx to replicate aerosolized respiratory particles during resuscitation, without the need for a human researcher to aerosolize the particles. Additionally, this emanation is contained within the mannequin by an appropriate bag-valve-mask seal. Future iterations of this mannequin may involve developing models that allow for the preservation of respiratory effort in the high-fidelity mannequin. This would allow for more realistic assessments for providers during scenarios.

A limitation of GloGerm powder includes its visualization under blacklight for trace areas of contamination. Dust particles and the GloGerm powder can look similar under blacklight, which may lead to either an overestimation or underestimation of contamination. While there are other tools to detect UV particles available on the market, the use of a blacklight is the most cost-effective method in this scenario.

As of the writing of this manuscript, we are unaware of other simulator models that allow for continuous aerosolization of a UV-luminescent powder while performing uninterrupted CPR resuscitation simulations. The cost of set-up for this mannequin is roughly $2940 (Laerdal Resusci Anne mannequin, Glogerm powder, oxygen tank, and Misty-May nebulizer). Prior set-ups have used air gun models, latex balloons, or attached syringe/bag-valve masks to simulate bursts. While these other set-ups vary in terms of their relative cost, they require an investigator to manually trigger bursts. This model is slightly more expensive than the continuous aerosolization model proposed by Matava et al. (priced at $2332) but offers the additional ability to perform cardiopulmonary resuscitation simulations during use. The development of GloGerm powder was intended for external use only, as a training aid to determine cross-contamination. Participants in this study were in full PPE, with surgical or N-95 masks, protective face shields, gowns, and gloves, to prevent mucosal exposure of the particulate matter. No hypersensitivity reactions or allergies were reported to this powder during our scenarios. This chemical is not considered hazardous by the 2012 Occupational Safety and Health Administration (OSHA) Hazard Communication Standard. GloGerm material data and safety information can be found on their website [18].

## Conclusions

Unlike task-trainer or airway-only simulators, this setup allows for full team-based resuscitations, creating a realistic cardiopulmonary resuscitation simulation during the COVID-19 pandemic. This allows for full team-based resuscitation simulations to occur using a particulate-generating mannequin without the interference of a human researcher. The ability to visualize particulate spread after the completion of resuscitation scenarios allows providers to reflect on various practice patterns and adequate PPE usage to prevent themselves most effectively from unnecessary exposure, preparing themselves for future scenarios. This construction of a simulated mannequin provides an affordable and effective method for healthcare teams to practice safe cardiopulmonary resuscitation methods in high-risk aerosolization scenarios.

## References

[1] Edelson DP, Sasson C, Chan PS (2020). Interim guidance for basic and advanced life support in adults, children, and neonates with suspected or confirmed COVID-19. From the Emergency Cardiovascular Care Committee and Get With The Guidelines-Resuscitation Adult and Pediatric Task Forces of the American Heart Association. Circulation.

[2] Morgan RW, Kienzle M, Sen AI (2020). Pediatric resuscitation practices during the coronavirus disease 2019 pandemic. Pediatr Crit Care Med.

[3] Chan PS, Berg RA, Nadkarni VM (2020). Code blue during the COVID-19 pandemic. Circ Cardiovasc Qual Outcomes.

[4] Lindsley WG, King WP, Thewlis RE, Reynolds JS, Panday K, Cao G, Szalajda JV (2012). Dispersion and exposure to a cough-generated aerosol in a simulated medical examination room. J Occup Environ Hyg.

[5] Lindsley WG, Reynolds JS, Szalajda JV, Noti JD, Beezhold DH (2013). A cough aerosol simulator for the study of disease transmission by human cough-generated aerosols. Aerosol Sci Technol.

[6] Matava CT, Yu J, Denning S (2020). Clear plastic drapes may be effective at limiting aerosolization and droplet spray during extubation: implications for COVID-19. Can J Anaesth.

[7] Oman SP, Helgeson S, Lowman P (2021). Novel repurposing of a Laerdal Airway trainer to simulate aerosolization. BMJ Simul Technol Enhanc Learn.

[8] Simpson JP, Wong DN, Verco L, Carter R, Dzidowski M, Chan PY (2020). Measurement of airborne particle exposure during simulated tracheal intubation using various proposed aerosol containment devices during the COVID-19 pandemic. Anaesthesia.

[9] Shavit D, Feldman O, Hussein K (2020). Assessment of alternative personal protective equipment by emergency department personnel during the SARS-CoV-2 pandemic: a simulation-based pilot study. Simul Healthc.

[10] Chanpong B, Tang M, Rosenczweig A, Lok P, Tang R (2020). Aerosol-generating procedures and simulated cough in dental anesthesia. Anesth Prog.

[11] Branecki CE, Jobeun NJ, Ronnfeldt TJ, Ash MA, Schulte TE, Langenfeld JG (2020). Novel barrier enclosure for both aerosol and droplet protection model. West J Emerg Med.

[12] Cheng A, Auerbach M, Hunt EA, Chang TP, Pusic M, Nadkarni V, Kessler D (2014). Designing and conducting simulation-based research. Pediatrics.

[13] DuCanto J, Barrix R (2020). Suction-assisted containment of respiratory droplets during airway management with the SALAD Technique. https://www.anesthesiologynews.com/Multimedia/Article/06-20/Suction-Assisted-Containment-of-Respiratory-Droplets-During-Airway-Management-With-the-SALAD-Technique/58676.

[14] Majid A, Ayala A, Uribe JP (2020). Protective strategies in a simulated model when performing percutaneous tracheostomies in the COVID-19 era. Ann Am Thorac Soc.

[15] Gardiner C, Veall J, Lockhart S (2020). The use of UV fluorescent powder for COVID-19 airway management simulation training. Anaesthesia.

[16] Patel SH, Yim W, Garg AK, Shah SH, Jokerst JV, Chao DL (2020). Assessing the physiological relevance of cough simulators for respiratory droplet dispersion. J Clin Med.

[17] Hallett S, Toro F, Ashurst JV (2021). Physiology, Tidal Volume. https://www.ncbi.nlm.nih.gov/books/NBK482502/.

[18] (2021). GloGerm safety data sheet. https://www.glogerm.com/sds/GGPSDS2021.pdf.

